# On the dynamics of a big drop in acoustic levitation

**DOI:** 10.1016/j.ultsonch.2023.106705

**Published:** 2023-11-24

**Authors:** Eduardo Cancino-Jaque, Josué Meneses-Diaz, Y. Vargas-Hernández, L. Gaete-Garretón

**Affiliations:** a Laboratorio de Ultrasonidos, Departamento de Física, Facultad de Ciencia, Universidad de Santiago de Chile, 917-0124, Avenida Victor Jara 3493, Estación Central, Santiago, Chile

**Keywords:** Acoustic levitation, Single drop, Non-linear acoustic

## Abstract

The acoustic levitation of a drop is a complex process that needs a high-intensity non-linear acoustic field; the sound pressure level has to be sufficient to raise the drop but not too large to avoid its atomization, limiting the maximum size of a levitated drop. In this paper, we present an experimental study of big drops levitation with a volume up to $\left(166\pm 2\right)\left[\mu l\right]$ and with an effective diameter $\left(6.82\pm 0.03\right)\left[\mathrm{mm}\right]$, figures one magnitude order larger than the maximum drop volume reported in the literature. Our acoustic levitator produces an acoustic field with a different shape than the field produced by a typical levitator. Our measurements and simulations of the acoustic field and drop dynamics suggest that the levitation of big drops is possible because the distribution of radiation pressure over the drop surface in our system differs from that in a typical acoustic levitator; its maximum value appears on the top surface of the drop and not in its equator. In addition, we determined the upper and lower limits of sound pressure necessary for the levitation of drops of various sizes that allow our system.

## Highlights

• An acoustic field with a particular shape can improve the levitation performance.
• The established limit of water drop size in acoustic levitation was overcome.
• A radiating plate can produce a different acoustic field for levitation.
• An acoustic field used for levitation can exhibit non-linear characteristics.

## 1 Introduction

Levitation is a technique to keep an object in suspension, free of contact with solid surfaces. This technique is used in disciplines where the sample characteristics could experience non-controlled changes when in contact with a container wall. These changes could affect the sample’s shape, its physical or chemical composition, or contaminate it [1], [2], [3]. For instance, the effect of the container on the alkalinity and pH stability in a sample has been reported [39]; also, the container effect on the synthesis of metal oxides has been observed [40].

Different techniques enable the levitation of objects, and each one has different advantages and disadvantages [4]. Among them, acoustic levitation in air is a versatile and suitable technique because the acoustic waves interact with solid and liquid materials with different shapes and sizes. Naturally, the levitation depends on the sample’s acoustic properties, such as density and sound speed [5]. Despite the advances in acoustic levitation, limitations still exist in the maximum size of levitated samples.

The acoustic levitation was used on samples of different natures, such as high-dense matter [6], objects with irregular shapes such as integrated circuits [7], and living animals [8]. Acoustic levitation has also been used for the measurement of different rheological properties, including density, surface tension, viscosity, and sound speed [9], [10]. Also, acoustic levitation has been utilized for studying phenomena such as eutectic growth in samples of high-density alloy [11] and the growth of ice particles [12]. In addition, Raman spectroscopy [13], X-ray crystallography [14], and other spectroscopic techniques have been used on samples on acoustic levitation.

When an object interacts with a high-intensity acoustic field, nonlinear effects appear. One is the radiation pressure, which produces a force over the object. If the force produced is strong enough to counteract gravity force, levitation is possible. Both progressive and standing acoustic fields can produce radiation pressure over an object, but the standing field is often used for acoustic levitation because it produces a force greater than a progressive field [15].

Different ways exist to produce acoustic levitation. One of them is to produce a stationary field using two in-phase synchronized transducers or a single transducer and a reflector, with a distance between them close to a multiple of half wavelength [5], [16]. Another way is to produce a pseudo-stationary wave using one or more arrays of transducers, with or without a reflector, and control each transducer’s amplitudes and phase shifts [17] with enough high precision. Replacing a plane reflector with a concave one increases the radiation force on the sample [18], [19], thus enhancing levitation possibilities.

Due to the dynamics of a drop, its acoustic levitation is more complex than the levitation of a solid sample. It depends on the drop’s rheologic properties and the acoustic field characteristics [20]. For the levitation of drops, the amplitude of the acoustic field must be kept between two limits: the lower limit is determined by the minimum pressure to overcome the gravitational force on the drop, and the upper limit should not be exceeded to avoid atomization of the drop.

Lee, Anilkumar, and Wang [21], [22], [23] experimentally studied the instability of a flattened drop in acoustic levitation and developed models to describe it. Using those results, Foresti et al.[24] obtained an expression for the critical acoustic Bond number to keep a drop in levitation before it atomizes. The acoustic Bond number describes the ratio between the radiation stress produced by the acoustic field over the drop and the drop’s surface tension.

Using this critical acoustic Bond number [24], Aoki and Hasegawa [25] calculated the maximum pressure $p_{\max}$ of the field for the stable acoustic levitation of a drop (1).

(1) $$p_{\max}=\sqrt{\sigma \rho_{0}c_{0}\left(\frac{3.2}{d}-\frac{1.3\pi }{\lambda }\right)}\text{.}$$

Here σ is the drop’s surface tension, $\rho_{0}$ is the medium density, $c_{0}$ is the sound speed, λ is the wavelength of the acoustic field, and *d* is the drop diameter. Considering all those factors, Aoki and Hasegawa predicted and corroborated that the maximum equivalent diameter of a water drop in acoustic levitation, with frequency of $19.3\left[\mathrm{kHz}\right]$, could not be bigger than 3.14[mm], corresponding to a volume of 16[μl]. The existence of this limit restrains the applications of acoustic levitation in both industrial and scientific applications. For instance, a minimum sample volume is necessary for measuring pH in a drop [41], it is of high interest the determination of pH in a pure liquid without contact with container walls [39]. Since in a sample, the mass scales with the cube of a typical length (radius) and the surface scales with a square of such magnitude in research on the volumetric properties of a free liquid, it is interesting to obtain large sample volumes to separate the surface properties from bulk properties. In addition, the availability of big drops allows a deeper study of the evolution of drops from volatile substances [42]. In an industrial application, for example, in the synthesis of a drug or reactive, requiring to be synthesized in an environment such as microgravity, acoustic levitation offers a simpler and more economical alternative.

In this paper, we report an experimental study of the dynamics of a water drop in acoustic levitation. Looking for conditions to maximize levitation performance, we tried different experimental configurations, resulting in the optimized system described in the following section. To the best of our knowledge, our system allowed us to achieve the levitation of drops with volumes at least one magnitude of order bigger than the maximum size reported in the literature [25]. In the following, we describe our axisymmetric levitator and characterize its acoustic field. Images of levitated drops of different sizes were acquired using a high-speed camera. Lower and upper Sound Pressure Level (SPL) limits for levitation for different volumes were found. Simulations of the field were made using the software COMSOL Multiphysics, and the results fit the experimental data well. Also, we show the differences between the acoustic field produced by a typical acoustic levitator and the acoustic field produced by our system. The radiation pressure acting on a levitated big drop was calculated using simulations, and the results allowed us to estimate the radiation pressure distribution over its surface, explaining why our system can levitate drops of such a size.

## 2 Acoustic levitator and experimental setup

### 2.1 Acoustic cavity for levitation

**Fig. 1:**
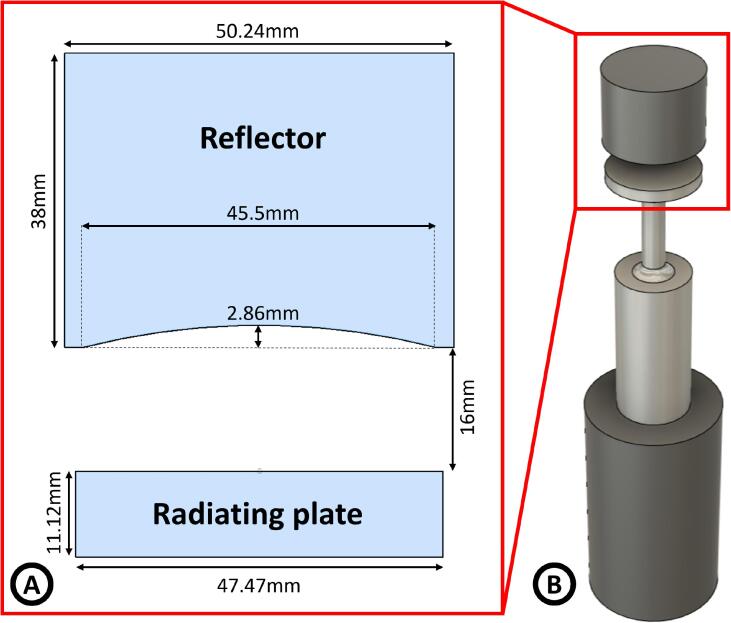
Scheme of the axisymmetric acoustic levitator. A) Dimensions of the acoustic cavity, radiating plate, and reflector. B) The draw of the levitator: reflector and electroacoustic stepped-horn transducer with the plate.

The acoustic levitator comprises an electroacoustic transducer with a radiating circular plate and a circular reflector. The plate is made of duralumin AA2017. The reflector is made of a stainless steel (316) cylinder with a spherical concavity carved on one of its faces. The curvature radius of the concavity is $92\left[\mathrm{mm}\right]$. Fig. 1A shows a schematic of the system, with the dimensions of the radiating plate and the reflector.

### 2.2 Transducer

An electroacoustic transducer, shown in Fig. 1B, excites the circular plate at its center. It consists of a prestressed piezoelectric sandwich-type transducer [26], followed by a stepped-horn-type mechanical amplifier [27]. The resonant frequency of the system is approximately 22.8[kHz], producing an acoustic field in the air with a wavelength close to 15 [mm].

The electronic excitation system consists of an electrical wave signal generator (Agilent 33220A) and a power amplifier AE TECHRON 7224. The transducer’s performance was monitored with a power meter ENI EMB 2K250. Because the feed current in the transducer correlates with the displacement of the transducer tip [28], the displacement was measured indirectly with a current probe Tektronix TCPA300.

The experimental signals were digitized and acquired with a scope (TiePie HS5) connected to a computer. The sampling frequency was at least ten times greater than the highest frequency relevant to the experiment. All signals were processed with MATLAB.s

### 2.3 Acoustic radiator

**Fig. 2:**
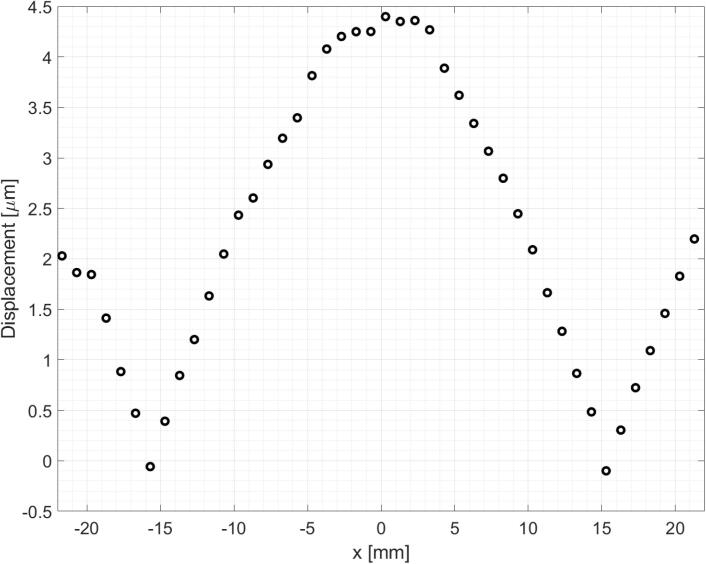
Radial scanning of the displacement amplitude of the circular radiating plate driven by the transducer with a constant feed current $\left(∼170\left[\mathrm{mA}\right]\right)$. It shows that the plate vibrates in its first flexural mode.

The radiating circular plate, driven by the transducer, oscillates in its first axisymmetric flexural vibration mode. Fig. 2 shows the results of a radial scanning of the vibrating plate measuring its out-of-plane displacement using a POLYTEC laser-DOPPLER system. The ratio between the displacement and the current (slope of displacement vs. current curve [28]) was $(25.7\pm 0.2)\left[\frac{\mu m}{A}\right]$, measured at the center of the plate.

### 2.4 Acoustic pressure measurement

The acoustic field was measured with a condenser microphone 1/8-inch type 4138 Brüel & Kjær (B&K). To protect the microphone, it was shielded with a special probe of 1.3[mm] diameter and 180[mm] length. We calibrated the probe by comparing the measurements by the protected and the non-protected microphone for the amplitude range from 60 to 150[dB], taking care not to exceed the maximum acoustic pressure level for the safe operation of B&K microphone. The measurements of SPL in the acoustic field were referenced to $20\left[\mu \mathrm{Pa}\right]$.

### 2.5 Image acquisition

The images of the drops were acquired with a Phantom Miro M310 camera. In each image, the resolution was approximately $60\left[\frac{\mu m}{\mathrm{px}}\right]$. The volume of the drops was calculated from the acquired images using MATLAB.

### 2.6 Experimental conditions

The experiments were done under a mean relative humidity of 43% and an atmospheric pressure of 955.6[mbar]; these parameters fluctuated less than 5%. The mean temperature was 20[°C] with fluctuations less than 30%.

## 3 Results and discussion

### 3.1 Acoustic Field

**Fig. 3:**
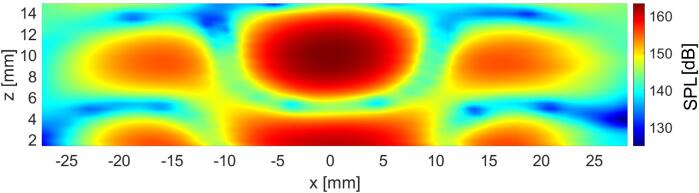
Sound pressure level of the fundamental frequency of the acoustic field in the XZ plane of the levitator.

A plotting of SPL measured between the radiating plate and the reflector in the XZ plane of the levitator is shown in Fig. 3.

**Fig. 4:**
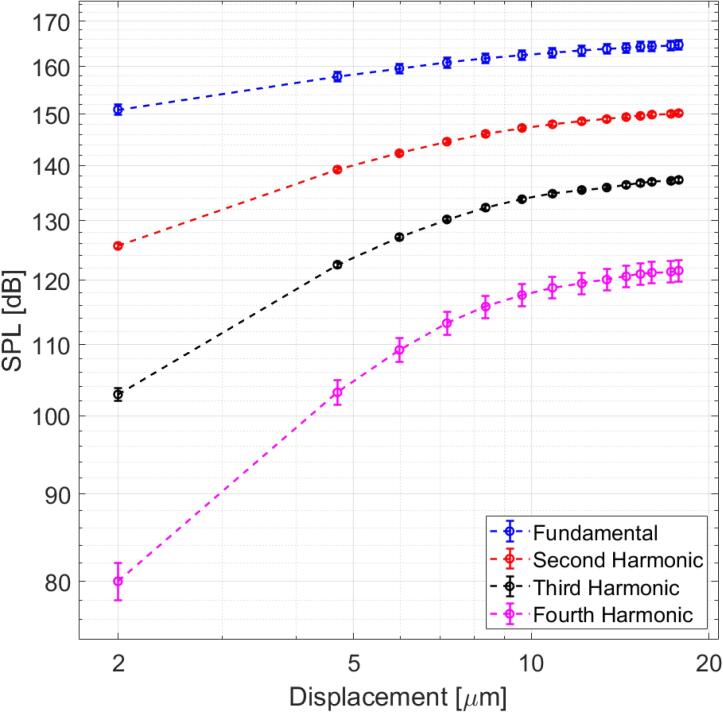
Sound pressure level (SPL) vs amplitude of the displacement. The SPL is measured in the region of maximum pressure and the displacement at the center of the plate.

Using the experimental procedure developed by Anilkumar et al. [22], the SPL was measured at the pressure antinode, located in $9.5\left[\mathrm{mm}\right]$ above the plate center. The antinode pressure was found to correlate with the amplitude of displacement. Fig. 4 shows the curves of SPL vs. the displacement amplitude of the plate center. The highly nonlinear character of the acoustic field is revealed by the high harmonic content of the waveform and the saturation tendency of the amplitude curves [29].

Nonlinearity in an acoustic levitator was described by Andrade et al. [33], who concluded, as it is well known in the high amplitude propagation studies, that the generation of harmonics in finite-amplitude waves is caused by the nonlinear propagation of the waves in air instead of their production by the source [34], [35], [29]. We verified the sinusoidal movement of the source in the regime studied; the amplitude of the higher harmonics was minor than 1% to the amplitude of the fundamental. A distorted stationary acoustic field produces additional forces over an object, which can be considerable when the object has millimeter dimensions [36], [44] and can be important for the capability of this acoustic field to maintain the drops in levitation. Forces produced by overtones of the field are called Oseen-type forces [44].

While the non-linear aspects can be important for drop levitation, the following will only consider the fundamental frequency of the field as it provides a sufficient description of the results of this study. Because the second harmonic detected in our nonlinear field is 15[dB] under the fundamental, it means that the effects of higher harmonics have not a significant effect on the considered phenomena.

Relevant aspects related to non-linear acoustic fields with a big drop, such as jump phenomenon [45], acoustic streaming [46], quantitative aspects of non-linear forces [44], and torques [47], will be considered in future research.

### 3.2 Acoustic Levitation of Drops

We used the setup described in Section 2 to produce the stable levitation of drops of distilled water.

**Fig. 5:**
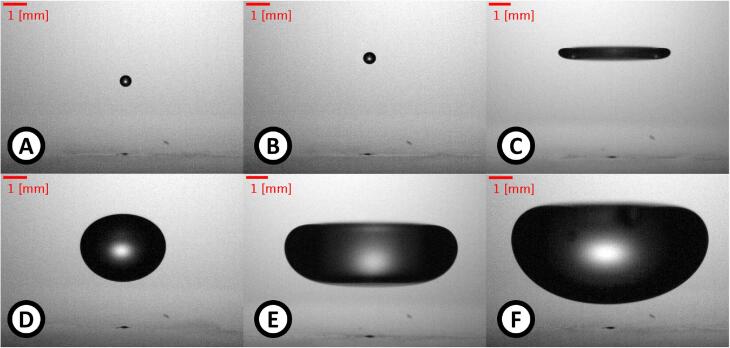
Stable levitation of drops of various sizes at different sound pressure levels. A) and B) volume: $\left(0.06\pm 0.01\right)\left[\mu l\right]$, SPL: $159.5\left[\mathrm{dB}\right]$ and $161.4\left[\mathrm{dB}\right]$, respectively. C) volume: $\left(7.8\pm 0.5\right)\left[\mu l\right]$ and SPL: $163.7\left[\mathrm{dB}\right]$. D) volume: $\left(19.39\pm 0.06\right)\left[\mu l\right]$ and SPL: $160.7\left[\mathrm{dB}\right]$. E) volume: $\left(108\pm 5\right)\left[\mu l\right]$ and SPL: $165\left[\mathrm{dB}\right]$. F) volume: $\left(166\pm 2\right)\left[\mu l\right]$ and SPL: $164.4\left[\mathrm{dB}\right]$. In each image, it is possible to see the center of the plate as a black spot under the drops.

The essential for levitation dynamics of a drop has been widely described in literature [3], [20], [21], [22], [23], [24], [25], [30], [31], [32]. However, the reported drop volume was always less than $20\left[\mu l\right]$. We levitated drops of small volume, for instance, in Fig. 5, Fig. 5, a $0.06\left[\mu l\right]$ levitated drop is shown at different SPL. Note that the relative position of a drop with respect to the vibrating plate center depends on the SPL. For instance, for SPL equal to 159.5[dB] (Fig. 5A) and 161.4[dB] (Fig. 5B), the drop position is different. In Fig. 5C, a levitated drop, with a volume of $\left(7.8\pm 0.5\right)\left[\mu l\right]$ and SPL $163.7\left[\mathrm{dB}\right]$, shows a shape flatter than a sphere; the high-intensity acoustic field distorts the shape of the drop.

Our study includes the typical size and shape of levitated drops, as shown in the previous paragraph. However, our main focus was to find the limits (if any) for the volume of levitated drops. Our analysis of the dynamics of drops suggested that with a suitable and non-conventional distribution of acoustic field pressures, it would be possible to produce and levitate drops with a volume bigger than the predicted maximum size limit [25].

**Fig. 6:**
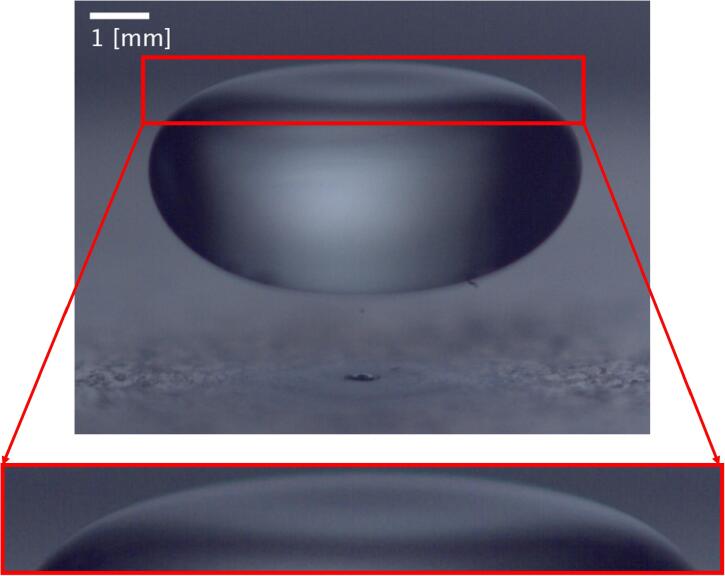
Image of a drop with the equatorial diameter greater than the capillary length. The red square shows a zoom made on the top surface of the drop. It shows a slight concavity on it.

Examples of big drops in levitation are shown in Figs. 5D, 5E and 5F. The biggest levitated drop, shown in Fig. 5F, had a volume of $\left(166\pm 2\right)\left[\mu l\right]$ and an effective diameter of $\left(6.82\pm 0.03\right)\left[\mathrm{mm}\right]$ $\left(∼\lambda /2.2\right)$. The maximum diameter of this drop is approximately 8.3[mm] $\left(∼\lambda /1.8\right)$ and its height is 4.2[mm] $\left(∼\lambda /3.6\right)$. When the equatorial diameter of a drop is larger than the capillary length (which in pure water is close to $2.7\left[\mathrm{mm}\right]$), the shapes of the top and bottom surfaces are different. In this case, the top surface can have a curvature that is not visible in a frontal image. An image taken from a different angle, shown in Fig. 6, helps to visualize this curvature.

**Fig. 7:**
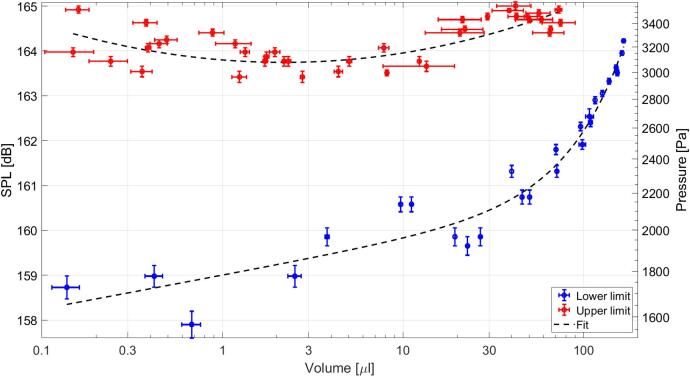
Limits found for acoustic levitation of drops with various volumes, lower limit (blue) and upper limit (red). Black segmented lines are the fits for each limit.

For the acoustic field shown in Fig. 3, we found the upper and lower limits of SPL for levitated drops of various sizes. The results for drops between $0.1\left[\mu l\right]$ and $166\left[\mu l\right]$ are plotted in Fig. 7. Note that SPLs shown in Fig. 7 correspond to the SPLs of the fundamental harmonic, shown in Fig. 4 by a blue curve. We found the upper limit of SPL increasing the displacement amplitude of the radiator center by $0.36\left[\mu m\right]$ steps until the drop atomized. For the lower limit, the displacement amplitude was decreased by $0.18\left[\mu m\right]$ steps until the drop fell.

**Fig. 8:**
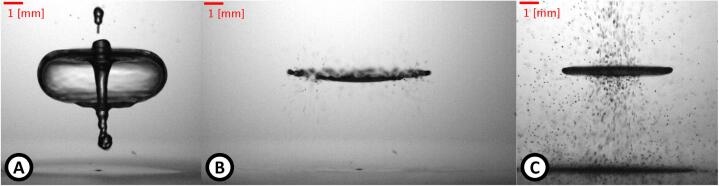
Images of atomization of a drop caused by instability and high SPL. A) Buckling instability. B) Kelvin–Helmholtz instability. C) Rayleigh-Plateau instability.

We also observed buckling, Kelvin–Helmholtz, and Rayleigh-Plateau instabilities of a levitated drop [21], Fig. 8.

Fig. 8A shows buckling instability that appears when the drop volume is high enough, making the influence of the surface tension, under the interaction of a strong acoustic field, become relativity weak. In this circumstance, the drop is transformed into a liquid sheet, and it has an upward motion, forming a closed membrane (or hollow shell), followed by the formation of a jet in the upper region and finishing with the atomization of the liquid. Buckling instability to transform a drop in a bubble has been reported [43].

Fig. 8B shows Kelvin–Helmholtz instability: when the acoustic field is high enough, but the drop volume is not so big, making surface tension still significant, the high particle velocity around the drop equator produces the flattening of it (pancake-type shape) and subsequent atomization by the circular border when the velocity reaches a critical value. Zang et al. [38] showed that when the equatorial diameter of the drop reaches a critical value, a positive feedback mechanism appears, producing the fast expansion of the drop.

Fig. 8C shows the Rayleigh-Plateau instability: when the acoustic field is high enough, capillary waves can be induced in the polar region of the drop. If SPL reaches a critical value, the capillary wave amplitude grows without limit, and the drop is atomized [21].

Of course, these instabilities for different liquids would depend on the whole rheological properties of the levitated drop.

**Fig. 9:**
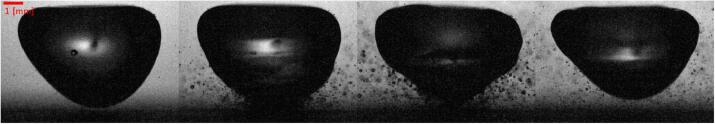
Sequence of images of a drop touching the plate when the pressure level is insufficient to maintain levitation. The time between frames is 8[ms].

For bigger drops, a curious phenomenon happens: the bottom of the drop may touch the vibrating plate, and a part of the drop atomizes. However, the drop does not entirely fall at the plate, and after losing some volume, a smaller drop remains in steady levitation. Fig. 9 shows a sequence of images of a drop exhibiting this phenomenon.

### 3.3 Numerical simulations

#### 3.3.1 Acoustic field

To learn more about the acoustic field in our levitator, we performed a finite element simulation of our system specified in Section 2 using the acoustic module of the COMSOL Multiphysics software. The properties of the medium (air) were speed of sound 343[m/s], density 1.2$\left[\mathrm{kg}/m^{3}\right]$, pressure 1[atm], temperature 20[°C], and the frequency was 22880[Hz]. We use a free triangular mesh with a minimum and maximum element size of $7.5\left[\mu m\right]\left(∼\lambda /2000\right)$ and $150\left[\mu m\right]\left(∼\lambda /100\right)$, respectively.

**Fig. 10:**
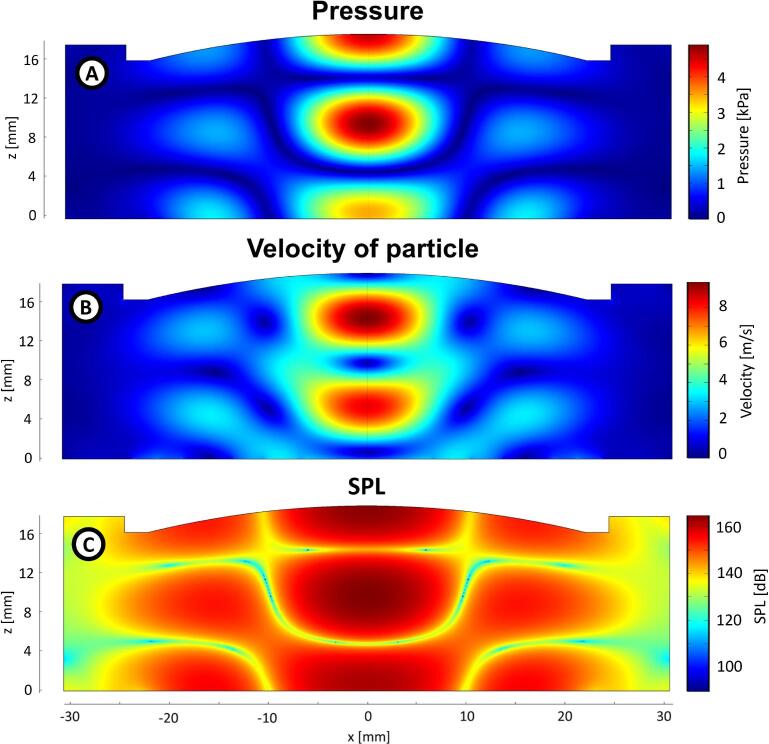
Result of the simulation of the acoustic field. A) Amplitude of the pressure. B) Amplitude of the velocity of the particle. C) SPL.

The results of the simulation are shown in Fig. 10: the amplitude of pressure (Fig. 10A), the amplitude of velocity of the particle (Fig. 10B), and SPL (Fig. 10C). Fig. 10C shows that the result of the simulation is very similar to the experimental data (Fig. 3), so we consider that the simulation correctly describes the acoustic field produced by this system.

The shape of the acoustic field used in this study (Fig. 3 and Fig. 10) is different from that in a typical levitator: there is a node region shaped like an axisymmetric “cup” or “bowl”. The morphology of this acoustic field is maintained if the distance radiator-reflector (specified in Fig. 1 decreases $∼0.5\left[\mathrm{mm}\right]$ $\left(∼\lambda /30\right)$ or increases $∼2.8\left[\mathrm{mm}\right]$ $\left(∼\lambda /5.5\right)$, decreasing the maximum SPL in 3[dB] and 5[dB] respectively. The shape of a stationary acoustic field is determined by different parameters of the system: characteristic of the radiator (shape and excitation frequency) and characteristics of the cavity (reflector geometry and distance radiator-reflector), being the boundary conditions the factors to define the resultant acoustic field. The use of a concave reflector complexes the resultant acoustic field, and it is necessary to employ numerical methods to find it, as has been described by Xie and Wei [16], and, seeing our results, the use of a vibrating plate also complicates it. This is the reason why the morphology of the acoustic field usually is not studied for levitation.

**Fig. 11:**
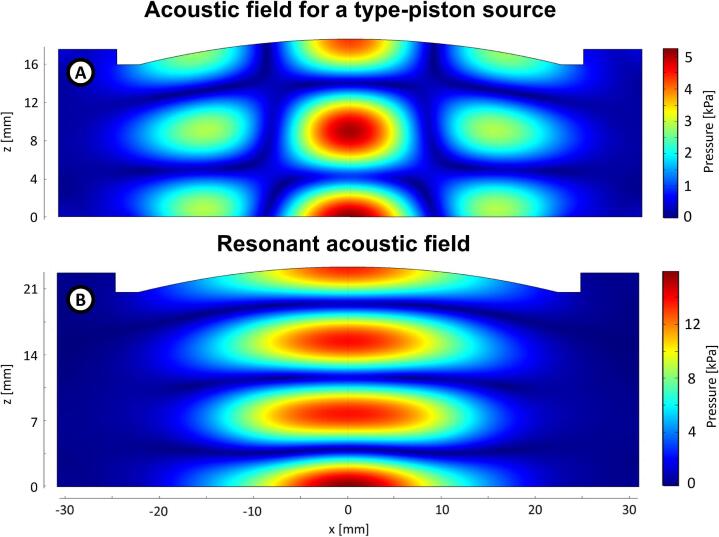
Simulations of the acoustic pressure field in a levitator. A) Acoustic levitator with the characteristics described in Fig. 1, but with a piston as a source. B) Acoustic levitator with a resonant distance between the radiator and the reflector.

The configuration of our particular experimental setup is very specific. If we replace, for instance, our flexing vibrating plate with a piston-like source, the shape of the acoustic field changes as it is shown in the simulation (Fig. 11A). Also, the dimensions used in the system play an essential role in the levitation process: if the distance between the radiating plate and the reflector is decreased or increased, the shape of the acoustic field changes. For instance, by increasing the source-reflector distance enough, the system reproduces a typical standing field shape used for acoustic levitation [5], [7], [33], [37], (Fig. 11B). We think it is produced by two factors, one of scale and another geometrical. The first one because at large distances, the shape of the reflector became less significant. The curvature of the reflector produces a curved negative step with a maximum depth of 2.8[mm], and the distance radiator-reflector is 21[mm]. It behaves like a plane reflector at 3λ/2 of the distance, acting like a resonant cavity; it’s a scale problem. The second factor is the directivity of the radiator; at several length-waves of distance, the contribution of the outer parts of the radiator shifted 180[°] from the center, does not reach the reflector, enhancing the contribution of the center of the radiator acting as a piston-like source, it’s a geometrical factor.

#### 3.3.2 Drop levitation and radiation pressure

The size of the drops reported in this paper is several times larger than what is expected and has been achieved thus far. We hypothesized that it is due to the specific distribution of acoustic field that differs from a typical standing wave, generally used to produce levitation.

The radiation pressure $p_{\mathrm{rad}}$ over the surface of a levitating drop can be calculated using Eq. 2, and the force $\overset{\to }{F}$, shown in Eq. 3, is the integral of the radiation pressure over the complete surface of the drop $S_{0}$.

(2) $$p_{\mathrm{rad}}=\frac{1}{2\rho_{0}c_{0}^{2}}\langle p^{2}\rangle -\frac{\rho_{0}}{2}\langle v^{2}\rangle$$

(3) F→=-∫S0pradn^dS

The radiation pressure over an object depends on the parameters involved in the process: the air properties (density $\rho_{0}$ and speed of sound $c_{0}$) and the characteristics of the acoustic field: the pressure *p* and velocity of the particles *v*.

Using experiments and simulations, Andrade and Marzo [30] investigated the significance of terms in Eq. 2 for a drop with an oblate shape in acoustic levitation. The first term, related to the pressure, is mainly responsible for the levitation, and the second term, related to the velocity of the particle, produces a suction force at the equator of the drop under the action of the Bernoulli effect, causing the flattening of it. When the second term exceeds a certain limit, the drop atomizes [31]. A similar conclusion was reached by Zhang et al. [38].

Following Andrade and Marzo [30] and Zhang et al. [38], we studied and compared the radiation pressure over the surface of a big drop as well as a flattened drop. The shape of the drops and their positions were obtained from the pictures shown in Fig. 5C and 5F. Using the same geometry of the simulation described in Section 3.3.1, we have placed the drops where their levitation occurs. The mesh was finer than the mesh used in Section 3.3.1 because we needed uniform element sizes on the surface of the drop. The element size was $35\left[\mu m\right]$ $\left(∼\lambda /446\right)$.

**Fig. 12:**
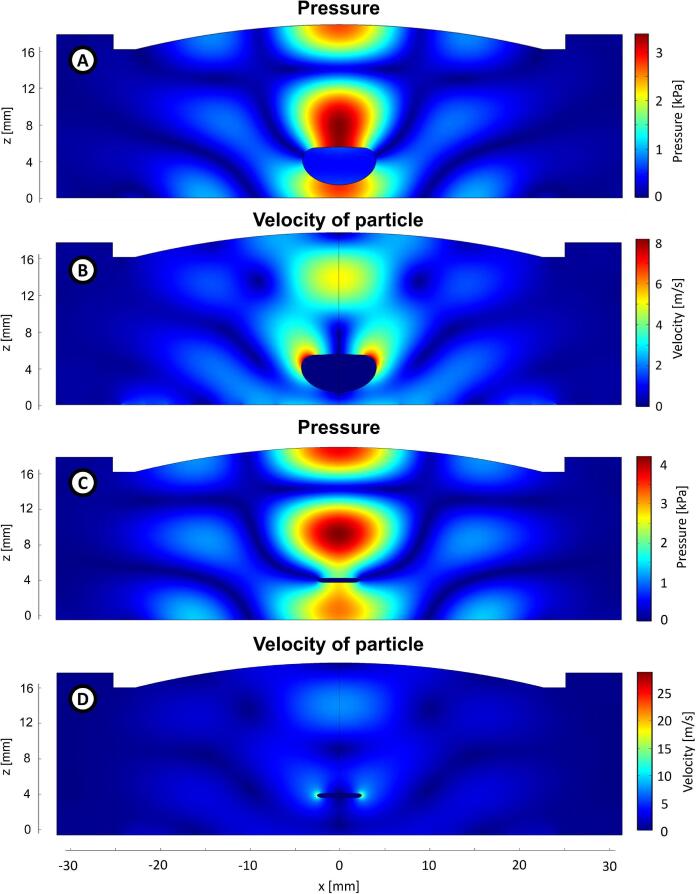
Results of the simulations of the acoustic fields with the drop shown in Fig. 5F (A and B) and flattened drop showed in Fig. 5C (C and D). In each case, the amplitude of pressure (A and C) and particle velocity (B and D) are shown.

The results are shown in Fig. 12. Those results indicate that the drop induces noticeable changes in the acoustic field: the pressure amplitude in both cases decreases; however, velocity amplitude increases with the flattened drop and decreases with the big drop. Their spatial distribution also changed, especially that of the particle velocity. The maximum amplitude of the particle velocity for the big drop is now on its top lateral surface.

**Fig. 13:**
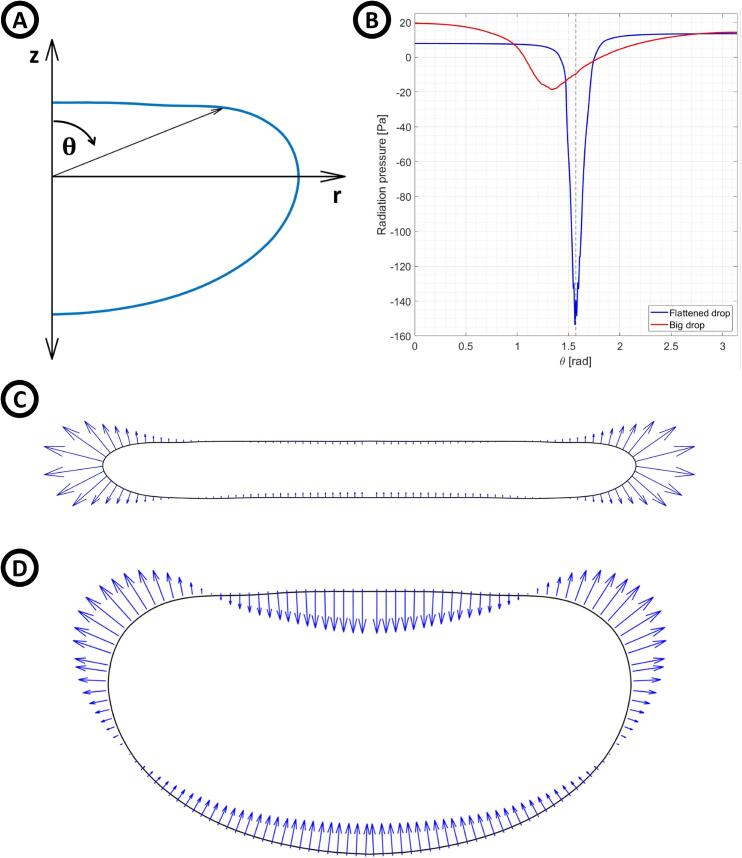
Flattened and big drop in acoustic levitation. A) Angle θ, used to scan the drop’s surface. B) Radiation pressure vs polar angle for both drops. The dashed black line shows the angle $\pi /2\left[\mathrm{rad}\right]$. C) and D) Drop profile and the radiation pressure (blue arrows) shown over the surface of the flattened drop (C) and the big drop (D).

To calculate the radiation pressure on the drops, the axial symmetry of the system allows scanning the complete drop’s surface with the polar angle 0⩽θ⩽π as shown in Fig. 13A. Using the data from the simulation shown in Fig. 12 and Eq. 2, the radiation pressure over the drop’s surfaces was calculated. Like Andrade and Marzo [30] and Zhang et al.[38], the maximum radiation pressure on the flattened drop, produced by the second term in Eq. 2, appears at $\pi /2\left[\mathrm{rad}\right]$ (Fig. 13B, blue curve). However, in the big drop it appears at a smaller angle (Fig. 13B, red curve). Figs. 13C and 13D show the radiation force distribution over the drop’s surface of the flattened drop and the big drop, respectively. Figs. 13B and 13D show that the suction force, generally acting at the equator of the drop surface (Fig. 13C), is now at the top of its surface.

The radiation pressure shown in Fig. 13B and 13D indicates that a component of the force that typically causes flattening of the drop now helps to levitate it. Eq. 2 provides another argument: in our case, both terms help to balance the gravitational force, while the responsible component of the flattening of the drops is weaker, allowing the levitation of drops bigger than a drop in a typical acoustic levitator.

## 4 Conclusion

We report an experimental study of acoustic levitation of a drop of distilled water with a volume larger than the reported maximum volume by at least one order of magnitude using a single acoustic source.

Our experiments and computer simulations reveal that the features of the acoustic field produced by our system allow for the levitation of big drops. In particular, the term in the radiation pressure related to the particle speed, which typically has its maximum over the drop equator and is responsible for its flattening and atomization, now has its maximum value on the top surface. As a result, this term helps to overcome the gravitational force, and its effect in the flattening of the drop with the increase of SPL is minor, thus allowing the levitation of big drops.

In addition, our experiments indicate that the upper limit of SPL necessary for acoustic levitation of drops is near a constant, independently of the volume. However, Eq. 1 indicates that the upper limit decreases when the volume is increasing. We think that Eq. 1 is only valid for drops levitated with classic equipment producing a planar nodal region. Otherwise, we have a nodal region with the shape of a “cup” or “bowl”. In other words, this classic model is not useful in our case.

In summary, our findings stress the importance of describing aspects of the acoustic field as its spatial distribution and usually are not considered in the investigation of acoustic levitation of drops.

## CRediT authorship contribution statement

**Eduardo Cancino-Jaque:** Conceptualization, Methodology, Software, Validation, Formal analysis, Investigation, Data curation, Writing - original draft, Writing - review & editing, Visualization, Funding acquisition. **Josué Meneses-Diaz:** Conceptualization, Methodology, Software, Validation, Formal analysis, Investigation, Writing - review & editing, Funding acquisition. **Y. Vargas-Hernández:** Conceptualization, Methodology, Resources, Writing - review & editing, Visualization, Project administration, Funding acquisition. **L. Gaete-Garret**ó**n:** Conceptualization, Methodology, Resources, Writing - review & editing, Visualization, Supervision, Funding acquisition.

## Declaration of Competing Interest

The authors declare the following financial interests/personal relationships which may be considered as potential competing interests: Eduardo Cancino-Jaque reports financial support was provided by National Agency for Research and Development (ANID) Scholarship National Doctoral Program No. 21190785. Josue Meneses-Diaz reports financial support was provided by National Agency for Research and Development (ANID) Scholarship National Doctoral Program No. 21200670.
